# TLR7 activation by imiquimod worsens glycemic control in female FVB/N mice consuming a high‐fat diet

**DOI:** 10.14814/phy2.15949

**Published:** 2024-02-12

**Authors:** Rahul M. Kakalij, Del L. Dsouza, LiGyeom Ha, Erika I. Boesen

**Affiliations:** ^1^ Department of Cellular & Integrative Physiology University of Nebraska Medical Center Omaha Nebraska USA

**Keywords:** autoimmunity, hyperglycemia, imiquimod, metabolic syndrome, toll‐like receptor 7

## Abstract

Toll‐like receptor‐7 (TLR7) activation promotes autoimmunity, and metabolic syndrome (MetS) is a common comorbidity in patients with autoimmune disease. We previously demonstrated hyperinsulinemia in TLR7 agonist imiquimod (IMQ)‐treated, high‐fat diet (HFD)‐fed female C57BL/6 mice. Since mouse strains differ in susceptibility to MetS and target organ damage, this study investigated whether 12 weeks of exposure to HFD and IMQ promoted MetS, autoimmunity, and target organ damage in female FVB/N mice. Supporting early‐stage autoimmunity, spleen‐to‐tibia ratio, and anti‐nuclear antibodies (ANA) were significantly increased by IMQ. No significant effect of IMQ on urinary albumin excretion or left ventricular hypertrophy was observed. HFD increased liver‐to‐tibia ratio, which was further exacerbated by IMQ. HFD increased fasting blood glucose levels at the end of 12 weeks, but there was no significant effect of IMQ treatment on fasting blood glucose levels at 6 or 12 weeks of treatment. However, oral glucose tolerance testing at 12 weeks revealed impaired glucose tolerance in HFD‐fed mice compared to control diet mice together with IMQ treatment exacerbating the impairment. Accordingly, these data suggest TLR7 activation also exacerbates HFD‐induced dysregulation of glucose handling FVB/N mice, supporting the possibility that endogenous TLR7 activation may contribute to dysglycemia in patients with autoimmune disease.

## 1 INTRODUCTION

Toll‐like receptors (TLRs) are essential to the innate immune system and are expressed on the cell surface or intracellular components of all innate immune cells. The ligands for TLRs are very broad, ranging from small‐size compounds to macromolecules and hydrophilic nucleic acids to hydrophobic lipids (Wang et al., 2016). TLRs can recognize pathogen‐associated molecular patterns (PAMPs) and damage‐associated molecular patterns (DAMPs) derived from damaged tissue (El‐Zayat et al., 2019). The binding of PAMPs and DAMPs to TLRs triggers specific intracellular signaling cascades that initiate host defense reactions; however, when inappropriately regulated, it leads to cellular damage. Although best known for their role in innate immunity, TLRs may also contribute to obesity‐induced insulin resistance and metabolic inflammation (Thomalla et al., 2022). Among all TLRs, TLR2 and TLR4 are the most studied TLRs in the context of insulin resistance, obesity, and glucose intolerance‐associated inflammation (Jialal et al., 2014). Evidence is also accumulating to suggest that TLR7 may play a role in several features of the metabolic syndrome (MetS).

Evidence for a role for TLR7 in MetS comes from both human and animal studies. TLR7 expression in subcutaneous adipose tissues is upregulated in human obesity, followed by increased expression of MyD88 and IRAK1 (Sindhu et al., 2015) and may also contribute to features of MetS. Consistent with endogenous activation of TLR7 occurring and playing a role in the development of MetS, TLR7 and TLR7/8 knockout mice were reported to have improved glucose tolerance tests, insulin tolerance tests, and reduced fat pad mass compared to wild‐type mice in the setting of diet‐induced obesity (Hanna Kazazian et al., 2019; Revelo et al., 2016).

A potential role for TLR7 activation in promoting MetS is of particular interest due to evidence that increased TLR7 activation may contribute to the development of the autoimmune disease lupus (Satterthwaite, 2021; Wang et al., 2022; Wolf et al., 2018), and that lupus is also associated with a high prevalence of MetS (Mok, 2019). Lupus is a systemic autoimmune disease predominantly affecting women (Beeson, 1994), and often accompanied by damage to the kidneys (lupus nephritis) and hypertension. Several experimental models of lupus exist, with a more recently described model being topical treatment with imidazoquinoline derivatives such as imiquimod (IMQ), which are recognized by TLR7 (Hemmi et al., 2002; Yokogawa et al., 2014). Although the original study by Yokogawa et al. (2014) did not report any metabolic parameters in IMQ‐treated mice that were maintained on regular rodent chow, we recently reported that combining IMQ and a high‐fat diet (HFD) for 6 weeks increased fasting blood glucose and insulin in female C57BL/6J mice (Kakalij et al., 2022). However, the autoimmune phenotype of these mice was mild in our hands, and unpublished data from our lab did not reveal substantial target organ damage in the C57BL/6 background, thereby limiting the model's usefulness. Yokogawa et al. (2014) also demonstrated the induction of autoimmunity in FVB/N mice in response to IMQ treatment. Longer periods of HFD exposure (>8 weeks) may also promote more marked effects on glycemic control (Leonardi et al., 2020). Therefore, the present study tested whether a more prolonged IMQ treatment and HFD exposure period (12 weeks) in female FVB/N mice would promote features of MetS, autoimmunity and target organ damage.

## 2 MATERIALS AND METHODS

### 2.1 Animals

All experimental procedures were approved in advance by the University of Nebraska Medical Center's Institutional Animal Care and Use Committee. Lupus is a chronic autoimmune disease that affects more women than men and is characterized by a 9:1 female‐to‐male ratio (Rider et al., 2018). Hence, based on the sex bias, the present study was limited to female mice. Female FVB/N mice were purchased from Jackson Laboratories (Bar Harbor, ME, Cat# JAX:001800; RRID: IMSR_JAX:001800), and *n* = 6–8 per group were housed on corncob bedding on a 12‐h light/dark cycle. Commencing at 12 weeks of age, mice were fed either a high‐fat “Western” diet (HFD; fat 42% kcal, sucrose 34% kcal; Cat# TD88137, Teklad, USA) or a control diet (fat 12.6% kcal, sucrose 34% kcal; Cat# TD05230, Teklad, USA), which was a modified version of the TD88137 diet with matched sucrose level. Both diets contained casein as a protein source. Except when mice were housed in metabolic cages for 24 h urine collection, mice were group housed in standard micro isolator cages.

### 2.2 Topical treatment with imiquimod

Based on a previously established protocol (Yokogawa et al., 2014), mice were untreated or treated epicutaneously on the ear three times weekly for 12 weeks with the TLR7 agonist IMQ at 1.25 mg of 5% IMQ cream (Aldara, Valeant Pharmaceuticals, NJ). This dose is much lower than that used in models of psoriasis (e.g., 62.5 mg daily; Hawkes et al., 2017), and no visible skin irritation was observed.

### 2.3 Analysis of glycemic control

To test for hyperglycemia at 6 weeks and prior to tissue collection at 12 weeks, conscious fasting blood glucose level was measured by glucometer (ACCU‐CHEK®, Aviva plus) in blood collected by tail prick after a five‐hour fast. An oral glucose tolerance test (OGTT) was also performed in a subset of mice after 11 weeks of the treatment. Briefly, mice were fasted for 5 h prior to oral glucose administration by gavage (2 g glucose/kg body weight in deionized water), and blood glucose level was measured (as described above) prior to glucose administration (i.e., fasting) and at 15, 30, 60, and 120 min after.

### 2.4 Measurement of urinary albumin excretion

Every 2 weeks, 24‐h urine production, food, and water intake were determined by housing mice individually in species specific metabolic cages (Lab Products Inc., Seaford, DE) for 24 h, during which time mice had free access to food and water. Urine albumin concentration was measured by ELISA (Albuwell M, Cat# 1011, Ethos Biosciences Inc, Logan Township, NJ; detection range lower limit of 0.156 μg/mL), allowing for calculation of urinary albumin excretion rate.

### 2.5 Tissue collection

At the end of 12 weeks, all mice were euthanized via isoflurane overdose (VetOne, Boise, ID), followed by thoracotomy. A terminal blood sample was collected by cardiac puncture using a heparinized syringe and centrifuged at 10,000*g* for 5 min at 4°C, and the plasma was collected and frozen at –80°C until analysis. Liver, gonadal fat, left ventricle, and spleen were collected and weighed, and tibia length was measured.

### 2.6 Real‐time PCR analysis

Total RNA from liver, spleen, and left ventricular tissue was extracted using Trizol reagent (Invitrogen, Carlsbad, CA; Cat# 15596026) and reverse transcribed into cDNA using a reverse transcription kit (Qiagen, Valencia, CA; Cat# 205314). Commercially validated QuantiTect primer assays (Qiagen) and SYBR Green‐based Luna® Universal qPCR Kit (New England BioLabs Inc., Ipswich, MA; Cat# M3003) were used to measure expression of TLR7 (Cat# QT00251013) relative to GAPDH (Cat# QT01658692) in spleen and liver, and atrial natriuretic peptide (ANP; Cat# QT00250922) and B‐type natriuretic peptide (BNP; Cat# QT00107541) relative to 18S ribosomal RNA (Cat# QT02448075) in left ventricular tissue, using the StepOnePlus real‐time PCR system (Applied Biosystems). Relative gene expression was quantified by the 2^**‐ΔΔ**Ct^ method.

### 2.7 Measurement of autoantibodies

Total anti‐nuclear IgG antibodies (ANA) (Cat# 5210, Alpha Diagnostics International Inc., San Antonio, TX, USA) and anti‐dsDNA IgG concentrations (Cat# 5120, Alpha Diagnostics International Inc., San Antonio, TX, USA) were determined in terminal plasma samples using commercially available ELISA kits according to the manufacturers' instructions. Positive index was calculated for ANA as the net ELISA optical density (OD) means and for anti‐dsDNA as the concentration (U/mL) means +2 standard deviations of the control/non‐immune samples.

### 2.8 Statistical analysis

All data are expressed as mean ± standard deviation. Statistical analysis was performed using GraphPad Prism v. 9.1.0 (RRID:SCR_002798) using a two‐way analysis of variance (ANOVA) testing for the main effects of dietary intervention (*P*_Diet_) or IMQ treatment status (*P*_IMQ_), and the interaction between diet and IMQ treatment (*P*_Diet*IMQ_). Where a significant effect was observed, pairwise comparisons were made via Bonferroni post‐hoc test, correcting for multiple hypothesis testing; *p* ≤ 0.05 was considered statistically significant.

## 3 RESULTS

### 3.1 HFD increases body weight and gonadal fat

**FIGURE 1:**
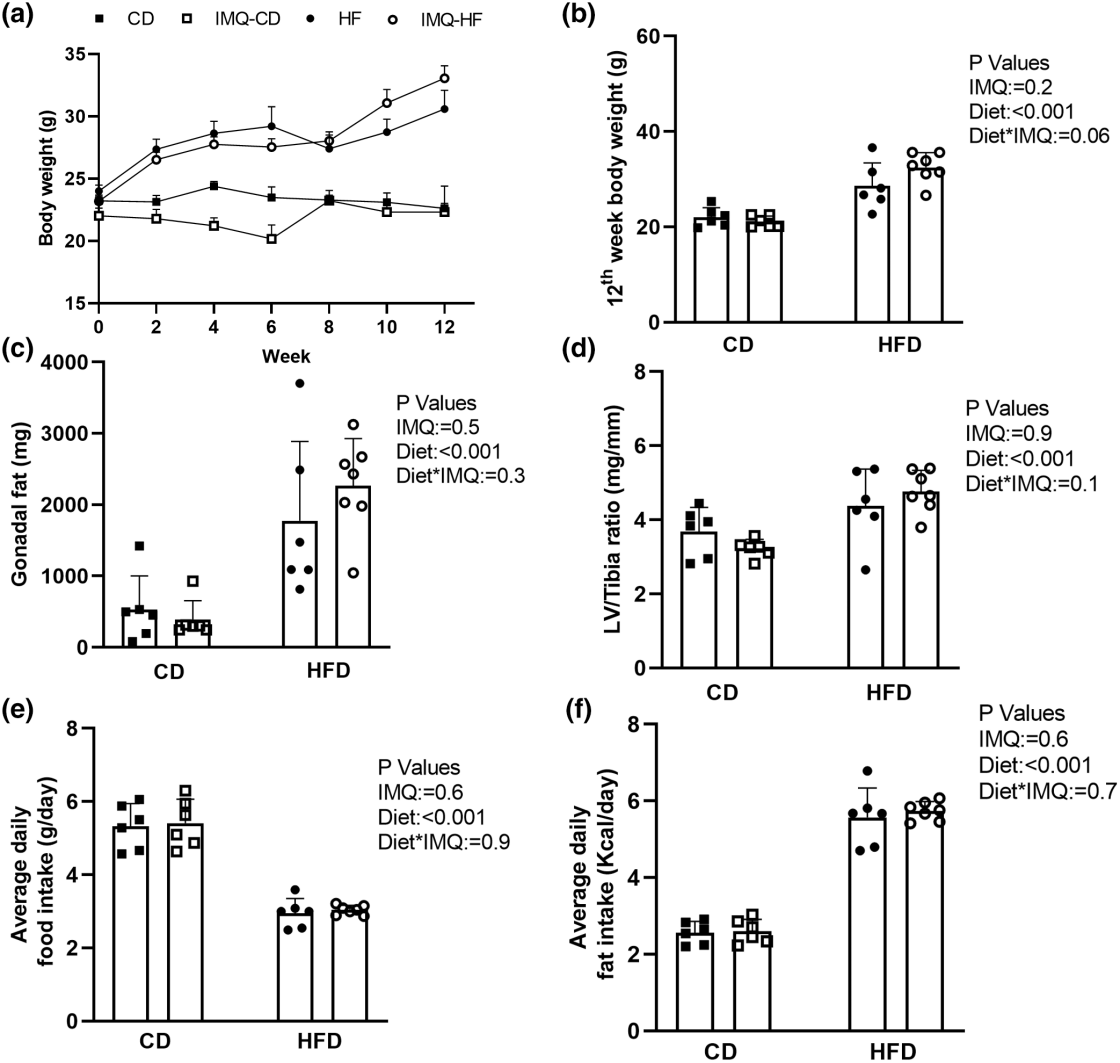
Female FVB/N mice underwent a 12‐week control diet (CD) or high‐fat diet (HFD) feeding protocol and were concurrently either treated or not with imiquimod (IMQ). (a) Body weight was recorded biweekly over the 12‐week study period. (b) Body weights at week 12 of the study. (c) Gonadal fat pad mass at week 12. (d) Left ventricle (LV)‐to‐tibia ratio at week 12. (e) Average daily food intake and (f) average daily fat intake during the 12 weeks were calculated based on food consumption measured biweekly for 24 h in metabolic cages. Data are presented as mean ± SD as well as individual data points for *n* = 6–8 mice per group, and analyzed by two‐way analysis of variance, testing for main effects of diet (*P*_Diet_), treatment group (*P*_IMQ_), or the interaction between the two (*P*_Diet*IMQ_). *P* values for post‐hoc comparisons were determined by Bonferroni testing.

Body weight was recorded every two weeks during the study period (Figure 1a). At the end of 12‐weeks, body weight was higher in HFD‐fed animals than in control diet‐fed animals (*P*_Diet_ < 0.001; Figure 1b). The response to diet was different depending upon whether mice were treated with IMQ (*P*_Diet*IMQ_ = 0.06; Figure 1b), with IMQ‐HFD mice weighing more than untreated HFD mice, while there was no difference between untreated and IMQ‐treated mice fed the control diet. Similar to the effect on body weight, gonadal fat pad mass was significantly greater in HFD‐treated mice than in control diet mice (*P*_Diet_ < 0.001; Figure 1c), but IMQ had no significant effect. Hypertension is an important feature of the MetS and often observed in lupus patients. As a first step towards testing whether any such hemodynamic disturbances or other cardiac pathology may have been induced by IMQ, with or without HFD exposure, left ventricle‐to‐tibia ratio was measured in all 4 groups. The left ventricle‐to‐tibia ratio was significantly elevated among HFD‐fed animals when compared with control diet‐fed animals (*P*_Diet_ < 0.001), but there was no significant effect of IMQ treatment (*P*_IMQ_ = 0.9; Figure 1d). There was also no significant difference in left ventricular ANP or BNP expression between groups (relative ANP expression using the 2^‐ΔΔCt^ method was 1.38 ± 1.04 in control diet untreated, 2.44 ± 2.45 in control diet IMQ‐treated, 2.18 ± 2.05 in HFD untreated and 1.84 ± 1.27 in HFD‐IMQ‐treated mice, *P*_IMQ_ = 0.6, *P*_Diet_ = 0.8, *P*_Diet*IMQ_ = 0.3; relative BNP expression was 1.43 ± 1.12 in control diet untreated, 2.34 ± 1.81 in control diet IMQ‐treated, 1.69 ± 1.28 in HFD untreated and 3.08 ± 3.79 in HFD‐IMQ‐treated mice, *P*_IMQ_ = 0.2, *P*_Diet_ = 0.6, *P*_Diet*IMQ_ = 0.8). There was also no significant correlation between left ventricle‐to‐tibia ratio and either ANP or BNP expression (data not shown). Averaging the biweekly metabolic cage measurements of 24‐h food intake showed no significant effect of IMQ (*P*_IMQ_ = 0.6) but reduced intake by food weight in HFD groups (*P*_Diet_ < 0.001; Figure 1e). Average daily fat intake calculated from this food intake data showed fat intake was significantly greater in HFD‐fed mice than control diet‐fed mice (*P*_Diet_ < 0.001) but was not significantly affected by IMQ treatment (*P*_IMQ_ = 0.6; Figure 1f).

### 3.2 No significant effect of IMQ or HFD on urinary albumin excretion

Urinary albumin excretion at 12 weeks averaged 40 ± 1 μg/day in control diet untreated, 31.8 ± 2 μg/day in control diet IMQ‐treated, 31 ± 2 μg/day in HFD untreated, and 26 ± 1 μg/day in HFD‐IMQ‐treated mice. There was no significant effect of either IMQ (*P*_IMQ_ = 0.4) or diet (*P*_Diet_ = 0.4) on albumin excretion, nor was there any significant interaction of diet and IMQ treatment (*P*_Diet*IMQ_ = 0.8).

### 3.3 Effect of HFD or IMQ on fasting blood glucose

**FIGURE 2:**
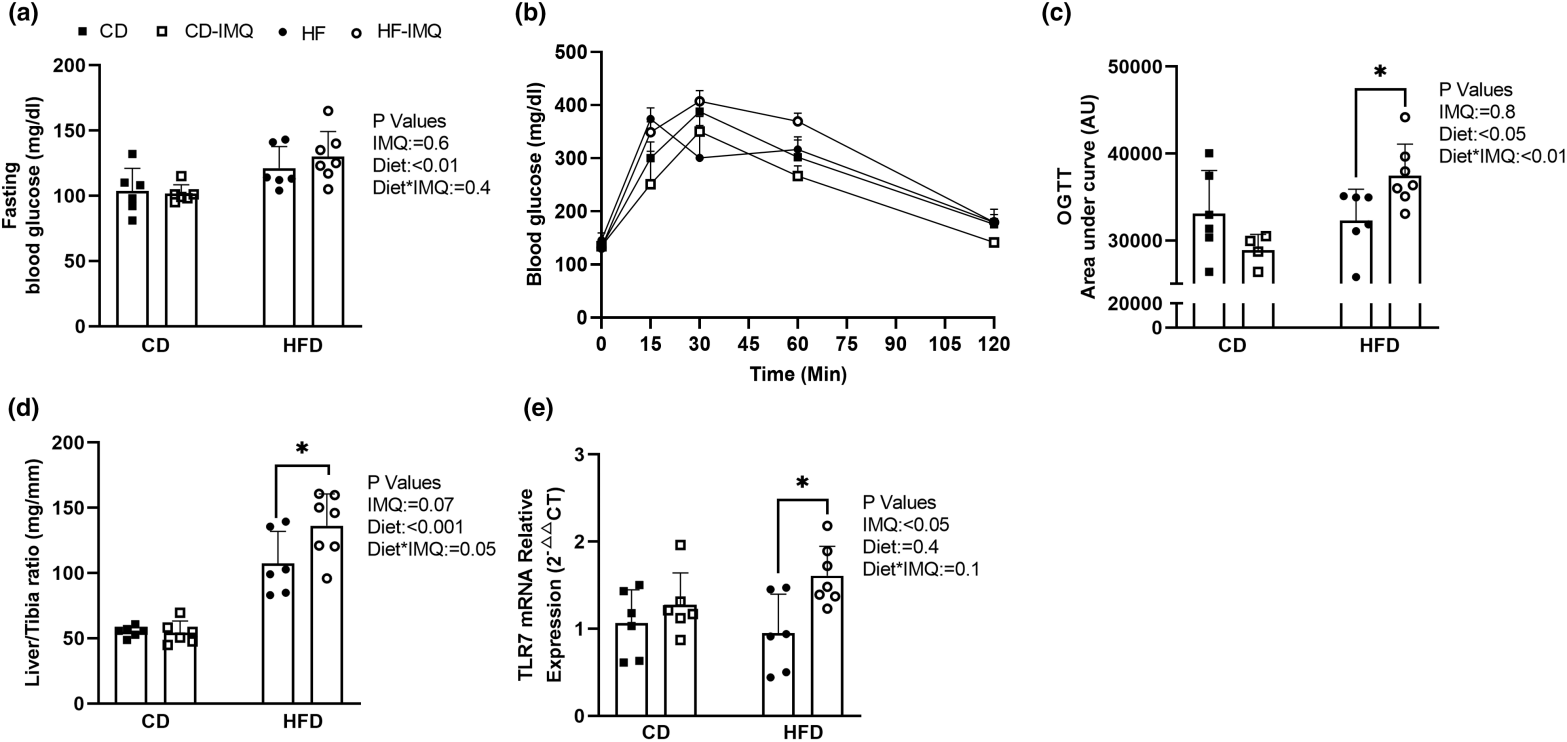
Glycemic control was evaluated in conscious, fasted mice at the end of the study period. Oral glucose tolerance testing (OGTT) was also performed in a subset of mice at week 11. (a) Fasting blood glucose measured prior to euthanasia and tissue collection at the end 12 weeks. (b) For OGTT, blood glucose measurements were measured before (time = 0) and after an oral glucose load. (c) The integrated area under the curve of OGTT results are depicted in (b) A.U. = Arbitrary Units. (d) Liver weight‐to‐tibia length ratio at 12 weeks. (e) Liver expression of TLR7 mRNA relative to GAPDH. Statistical analysis was performed as per Figure 1, and data are presented as mean ± SD and individual data points for *n* = 4–7 mice per group.

Fasting blood glucose at 6 weeks was 143.8 ± 14 mg/dL in control diet untreated, 105 ± 2 mg/dL in control diet IMQ‐treated, 134.6 ± 3 mg/dL in HFD untreated and 136.3 ± 4 in HFD‐IMQ‐treated mice. At this time point, there was no significant effect of IMQ (*P*_IMQ_ = 0.3) or diet (*P*_Diet_ = 0.4) on fasting blood glucose, nor any significant interaction of diet and IMQ treatment (*P*_Diet*IMQ_ = 0.7). At the end of twelve weeks, fasting blood glucose was higher in HFD‐fed animals than in control diet‐fed animals (*P*_Diet_ < 0.01; Figure 2a) but there was no statistically significant effect of IMQ treatment (*P*_IMQ_ = 0.6).

### 3.4 IMQ treatment impairs oral glucose tolerance in a diet‐dependent fashion

To assess glucose handling more sensitively at week 11, mice underwent oral glucose tolerance testing (OGTT; Figure 2b). Impaired glucose tolerance, as indicated by the increased area under the curve (AUC) of the OGTT, was observed in response to HFD overall (*P*_Diet_ < 0.05), but this was apparently driven in a diet‐dependent fashion (*P*_Diet*IMQ_ < 0.01; Figure 2c) with a significant increase in AUC only in the HFD‐IMQ‐treated group compared with untreated HFD‐fed mice (*P* < 0.05; Figure 2c). There was no overall effect of IMQ alone (*P*_IMQ_ = 0.8), suggesting that only IMQ was unable to sensitize mice to the dysglycemia effects.

### 3.5 IMQ treatment accentuated HFD‐induced hepatomegaly and increases liver TLR7 expression

Liver‐to‐tibia ratio was significantly elevated among HFD‐fed animals when compared with control diet‐fed animals (*P*_Diet_ < 0.001; Figure 2d), as well as hepatic steatosis observed in hematoxylin and eosin‐stained liver sections from HFD mice (data not shown). Further, there was a significant effect of IMQ treatment to accentuate the hepatomegaly in HFD‐fed mice (*P*_Diet*IMQ_ = 0.05; Figure 2d). Liver TLR7 mRNA was similarly increased by the combined IMQ and HFD exposure (Figure 2e).

### 3.6 IMQ increased spleen weight and plasma ANA but not anti‐dsDNA antibodies

**FIGURE 3:**
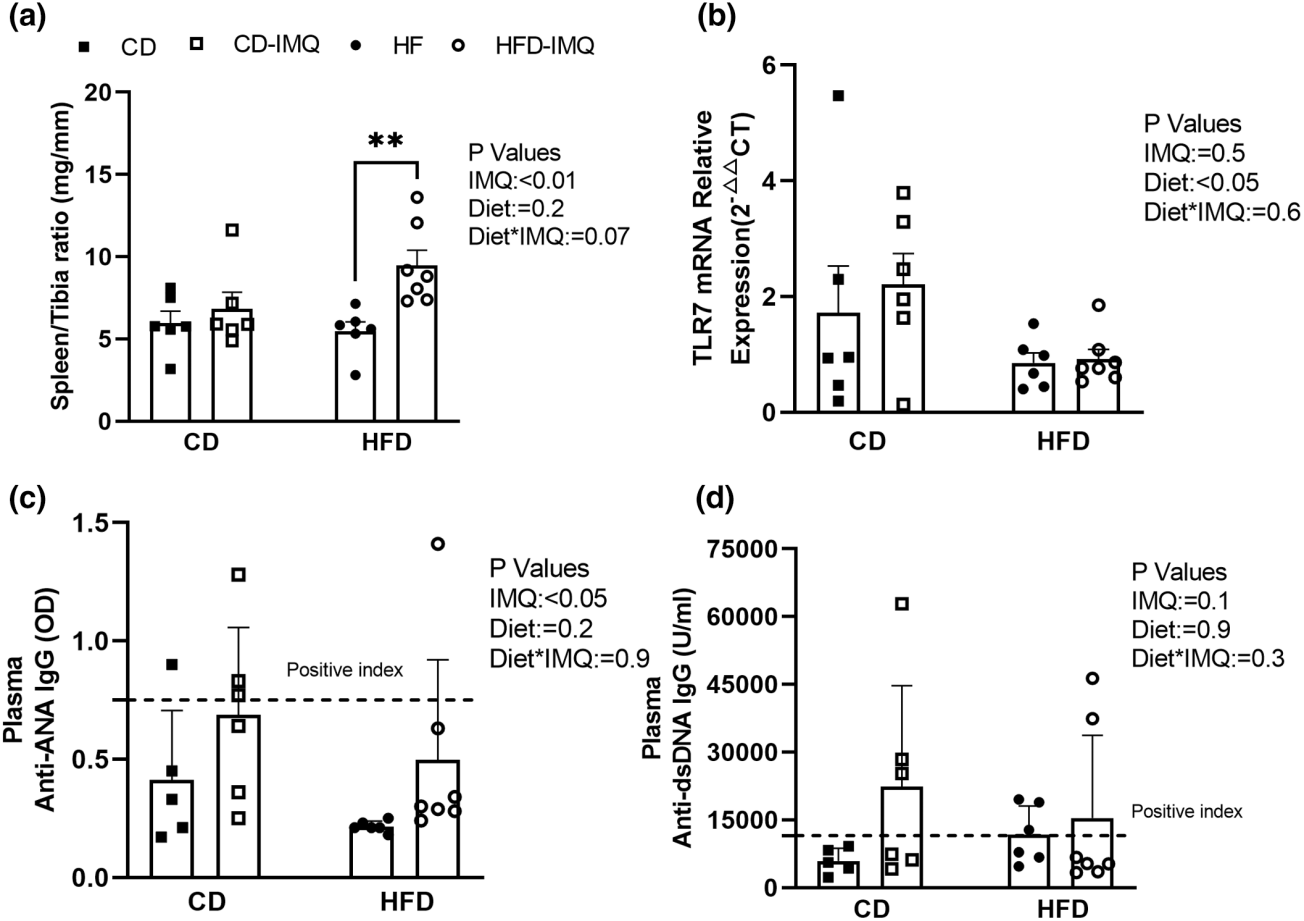
Assessment of autoimmunity after 12 weeks. (a) Spleen weight‐to‐tibia length ratio increased in IMQ‐treated mice overall (*P*_IMQ_ <0.01). (b) Spleen expression of TLR7 mRNA relative to GAPDH. (c) Levels of plasma anti‐nuclear IgG antibodies (ANA) expressed as the optical density (OD) measured using an ELISA at a consistent dilution of all samples. The positive index (dashed line) was calculated as the net ELISA kit optical density (OD) mean + 2 standard deviations of the control diet untreated (non‐immune) group. (d) Plasma anti‐dsDNA IgG antibodies. Statistical analysis was performed as per Figure 1, and data are presented as mean ± SD and individual data points for *n* = 5–7 mice per group.

Spleen weights were variable but significantly greater in HFD‐fed IMQ‐treated mice, 178.4 ± 5 mg, compared to HFD‐fed untreated mice, 102.5 ± 2 mg (*P*_IMQ_ < 0.05). The spleen‐to‐tibia ratio was significantly elevated only in the HFD‐IMQ‐treated animals compared to untreated HFD‐fed mice (*P*_IMQ_ < 0.01 Figure 3a). TLR7 mRNA expression in spleen was variable, and although there was no significant effect of IMQ (*P*_IMQ_ = 0.5), HFD diet‐fed animals showed significantly reduced TLR7 expression relative to the control diet animals (*P*_Diet_ < 0.05, Figure 3b). The average plasma concentration of ANA was also significantly increased in IMQ‐treated mice compared to untreated mice (*P*_IMQ_ < 0.05; Figure 3c), although most individual mice remained below the level considered to be the positive index. Plasma anti‐dsDNA IgG concentrations were inconsistently elevated in a small number of mice (Figure 3d). Neither IMQ treatment (*P*_IMQ_ = 0.1) nor diet (*P*_Diet_ = 0.9) had a significant overall effect on anti‐dsDNA IgG concentrations (Figure 3d).

## 4 DISCUSSION

Not only are TLRs known for their role in innate immunity, but a role in the progression of obesity‐related inflammation and metabolic complications has also emerged. The current study, as well as recent work by our group (Kakalij et al., 2022) and others (Hanna Kazazian et al., 2019; Revelo et al., 2016) suggest that enhanced TLR7 activation may exacerbate the development of MetS features, including impaired glucose homeostasis and insulin resistance. We report that pharmacological TLR7 activation promoted impairment of glucose tolerance in female FVB/N mice exposed to a HFD for 12 weeks. In using an oral glucose challenge in the present study, it is possible that IMQ‐ and HFD‐induced alterations in incretins as well as insulin. Although we lacked sufficient plasma for measurements of insulin or incretin hormones in the present study, we have previously reported increased fasting insulin levels following 6 weeks of IMQ plus HFD‐treatment of female C57BL/6J mice compared to HFD alone (Kakalij et al., 2022). C57BL/6 mice are regarded as susceptible to developing MetS when placed on a hypercaloric diet (Leonardi et al., 2020). We did not detect a significant independent effect of IMQ on FVB/N fasting blood glucose in the current study at either 6 or 12 weeks, whereas there was a significant increase in C57BL/6J mice at 6 weeks (Kakalij et al., 2022), perhaps reflecting strain differences in the response to HFD. Revelo et al reported that 8 days of IMQ treatment impaired glucose tolerance but did not raise fasting blood glucose in male C57BL/6 mice maintained on a normal control diet (Revelo et al., 2016). Conversely, knockout of TLR7 appears protective against the effects of HFD, with male TLR7 knockout mice on a C57BL/6 background showing improved glucose tolerance, insulin signaling, and reduced fat pad mass compared to wild‐type mice (Revelo et al., 2016). Similar findings of protection from increased fat pad mass and improved glucose tolerance have also been reported in female TLR7/8 double knockout mice (Hanna Kazazian et al., 2019). In the current study, we did not detect any significant effect of IMQ on body weight or gonadal fat pad mass, whereas IMQ treatment attenuated the effect of HFD on these variables in female C57BL/6J mice in our previous study (Kakalij et al., 2022). We did not perform parallel experiments in male mice, but the work of others described above (Revelo et al., 2016) did study male mice. Together, these data suggest that TLR7 activation can impair glucose homeostasis in males and females, although effects of TLR7 on adiposity are less consistent.

Precisely how TLR7 would become activated in MetS isn't fully understood. TLR7 receptors localize to endosomes and recognize viral dsRNA, ssRNA, and hypomethylated dsDNA. One likely endogenous source of TLR7 agonists and autoantigens that has been implicated in lupus and other autoinflammatory diseases are neutrophil extracellular traps (NETs) (Loh & Lam, 2022). NETs are weblike extracellular extrusions produced by neutrophilic granulocytes, containing chromatin, DNA, and antimicrobial substances as part of the innate immune response (Loh & Lam, 2022). Interestingly, HFD‐induced obesity is associated with increased release and decreased clearance of NETs (Revelo et al., 2016). In our experimental context, we utilized IMQ cream as an exogenous TLR7 agonist. Because our studies did not include TLR7 knockout mice or a control unmedicated cream, we cannot rule out potential “off target” effects of this experimental treatment. Concerns about non‐specific effects of IMQ cream have been raised in the field of psoriasis, where models use much higher amounts (62.5 mg vs. 1.25 mg) and daily administration (Hawkes et al., 2017). Nonetheless, IMQ is well‐accepted in the literature as an immunoactivating TLR7 agonist, based on work by (Hemmi et al., 2002) showing lack of response of TLR7 deficient mice and macrophages derived from those mice, and normal TNF‐α generation by TLR2, TLR4, or TLR9 knockout mice. Whether HFD induced NET formation and consequently endogenous TLR7 activation also contributed to the effects observed in the present study remains an open question.

Low dose application of the TLR7 agonist IMQ treatment exerted a mild but significant effect to promote features of autoimmunity in FVB/N mice in the current study, including splenomegaly and significantly increased plasma ANA antibody levels, although these remained variable and below the positive index in many of the mice. Models of autoimmunity tend to show inherent variability, as indeed to autoimmune patients. Similarly to 6‐weeks of IMQ treatment of C57BL/6 mice in our previous study (Kakalij et al., 2022), the present study did not see a significant increase in anti‐dsDNA antibodies in IMQ‐treated mice. It has been reported that 12 weeks of topical IMQ exposure to NZBWF1 did not increase serum anti‐dsDNA antibodies (Hayakawa et al., 2022). In the 8‐week study conducted by Yokogawa et al. (2014), IMQ‐treated mice were reported to have increased plasma levels of anti‐dsDNA, anti‐Sm autoantibodies, and anti‐RNP antibodies. In another study by Yudong et al, a three‐week IMQ treatment promoted higher levels of serum total IgG, anti‐dsDNA, anti‐histone, and anti‐RNP/SM autoantibodies in B6/WT mice (Liu et al., 2018). Recently, Su et al reported hepato‐splenomegaly, ascites, increased urinary protein excretion, and serum anti‐dsDNA level in the FVB/N mice after 8 weeks of IMQ treatment (Su et al., 2022). We also observed an IMQ‐induced exacerbation of HFD‐induced hepatomegaly in our study but an overall milder phenotype than Su et al. As such, effects of IMQ on autoantibody levels and other phenotypes seem to vary between mouse strains and investigative teams, for reasons that are not readily apparent.

Several human and animal research studies suggest that obesity provides a favorable environment for developing autoimmune diseases and systemic inflammation (Kwiat et al., 2022; Matarese, 2023). Indeed, mice fed 60% kcal from fat diets for 15–18 weeks and obese humans have increased serum anti‐nuclear antibodies, possibly as a result of increased generation and reduced clearance of NETs as described above (Hanna Kazazian et al., 2019; Revelo et al., 2016). We did not detect any significant synergistic effects of a 42% kcal from fat HFD on the development of splenomegaly or autoantibodies in response to the TLR7 agonist in FVB/N mice in this 12‐week study, or in our previous 6‐week study of IMQ treatment of C57BL/6J mice (Kakalij et al., 2022). It is possible that a longer period of HFD feeding or higher percentage of fat may be necessary to potentiate autoantibody formation in non‐autoimmune strains of mice. The type and percentage of dietary fat may also influence autoantibody production and the progression of disease in mouse models of lupus, although even in autoimmune strains the effects of HFD to increase autoantibodies are not consistently seen (Alexander et al., 1987; Choi et al., 2022; Gilbert & Ryan, 2011; Kelley & Izui, 1983).

One goal of the current study was to determine whether autoimmune‐mediated target organ damage would be observed in IMQ‐treated FVB/N mice and exacerbated by HFD. In lupus nephritis, anti‐dsDNA antibody complexes bind to the components of the glomerulus, inducing inflammation and tissue damage, ultimately leading to increased urinary albumin excretion. We did not observe a significant effect of IMQ or HFD on urinary albumin excretion in the current study. Consistent with this, we also did not observe a significant increase in anti‐dsDNA antibodies, although concentrations were highly variable within groups. Although HFD animals showed an increased left ventricle weight‐to‐tibia length ratio, we did not detect any significant effect of IMQ treatment to exacerbate left ventricular hypertrophy, nor was there any significant effect of either treatment on left ventricular ANP or BNP expression. Similarly, no IMQ‐induced exacerbation of HFD‐induced left ventricular hypertrophy was observed in our previous study; indeed, HFD‐related ventricular hypertrophy appeared to be blunted by IMQ in C57BL/6 mice, for reasons that are not currently clear (Kakalij et al., 2022). The liver seemed to be a more sensitive target in this study, with increased liver‐to‐tibia ratio of IMQ‐treated HFD‐fed animals and upregulated TLR7 mRNA expression. The liver plays a key role in glucose homeostasis, and so we speculate that effects on the liver may have contributed to the apparent impairment glucose intolerance, although effects on other organs cannot be ruled out.

In conclusion, 12‐weeks of co‐treatment of TLR7 agonist IMQ with HFD exacerbated splenomegaly, hepatomegaly and impaired OGTT in female FVB/N mice. Our study contributes to a growing body of literature supporting the possibility that endogenous TLR7 activation may contribute to dysglycemia in the setting of obesity and perhaps in patients with autoimmune disease.

## AUTHOR CONTRIBUTIONS

RK and EB designed the experiments and drafted the manuscript. RK, DD, LG, and EB conducted the experiments, acquired, and analyzed the data. All authors participated in editing and finalizing the manuscript.

## FUNDING INFORMATION

The experiments were funded by the corresponding author's internal discretionary spending account/departmental lab funds. No grant funds were used.
